# Supplementary material for: De novo human genome assemblies reveal spectrum of alternative haplotypes in diverse populations
Source: Nat Commun. 2018 Aug 2;9:3040. doi: 10.1038/s41467-018-05513-w (PMC6072799; doi:10.1038/s41467-018-05513-w)

SUPPLEMENTARY INFORMATION

De novo human genome assemblies reveal spectrum of alternative haplotypes in diverse populations

Wong et al.

The Supplementary Information contains 4 Supplementary Figures, 3 Supplementary Tables, and 1 Supplementary Note.

SUPPLEMENTARY FIGURES

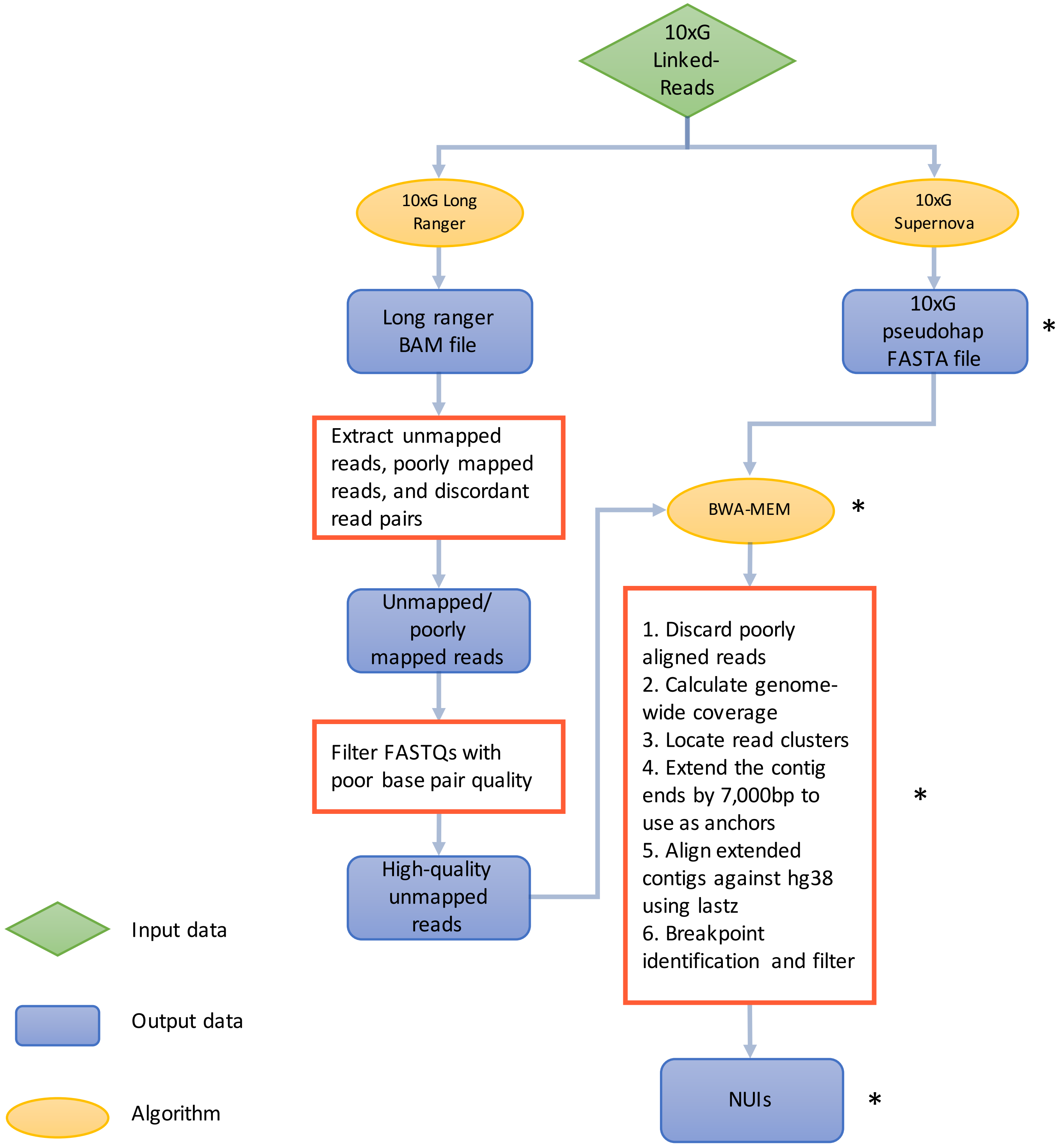

Supplementary Figure 1: Workflow depicting the non-reference unique insertions calling strategy. Steps that are performed twice (one for each pseudo-haplotype) are labeled with an asterisk. The entire pipeline was run on every individual, and the output from each sample was merged together to make a unified, non-redundant call set.

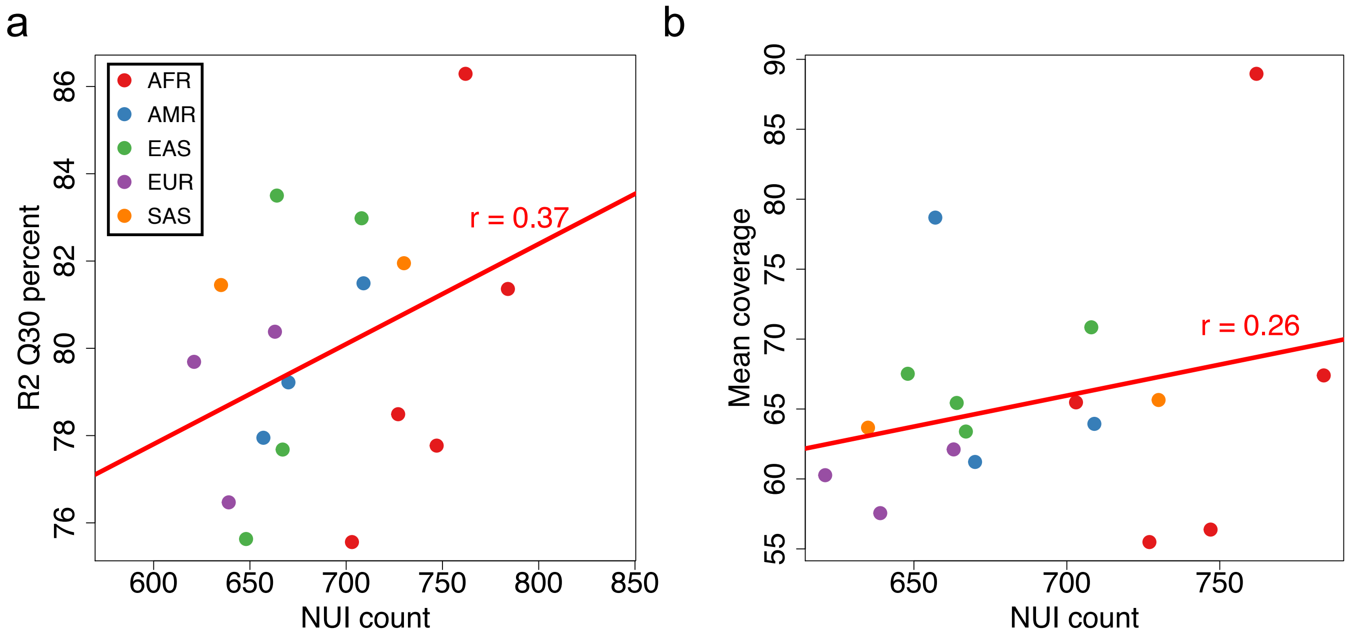

Supplementary Figure 2: Non-reference unique insertions quality control. (a) Scatter plot showing whether the NUI count was influenced by the quality of the sequencing reads. *R2 Q30* corresponds to the fraction of bases in read2 with a base quality ≥ 30 (Pearson's r = 0.37; p = 0.1414). (b) As in **a** but the mean coverage was plotted against the NUI count (Pearson's r = 0.26; p = 0.3159).

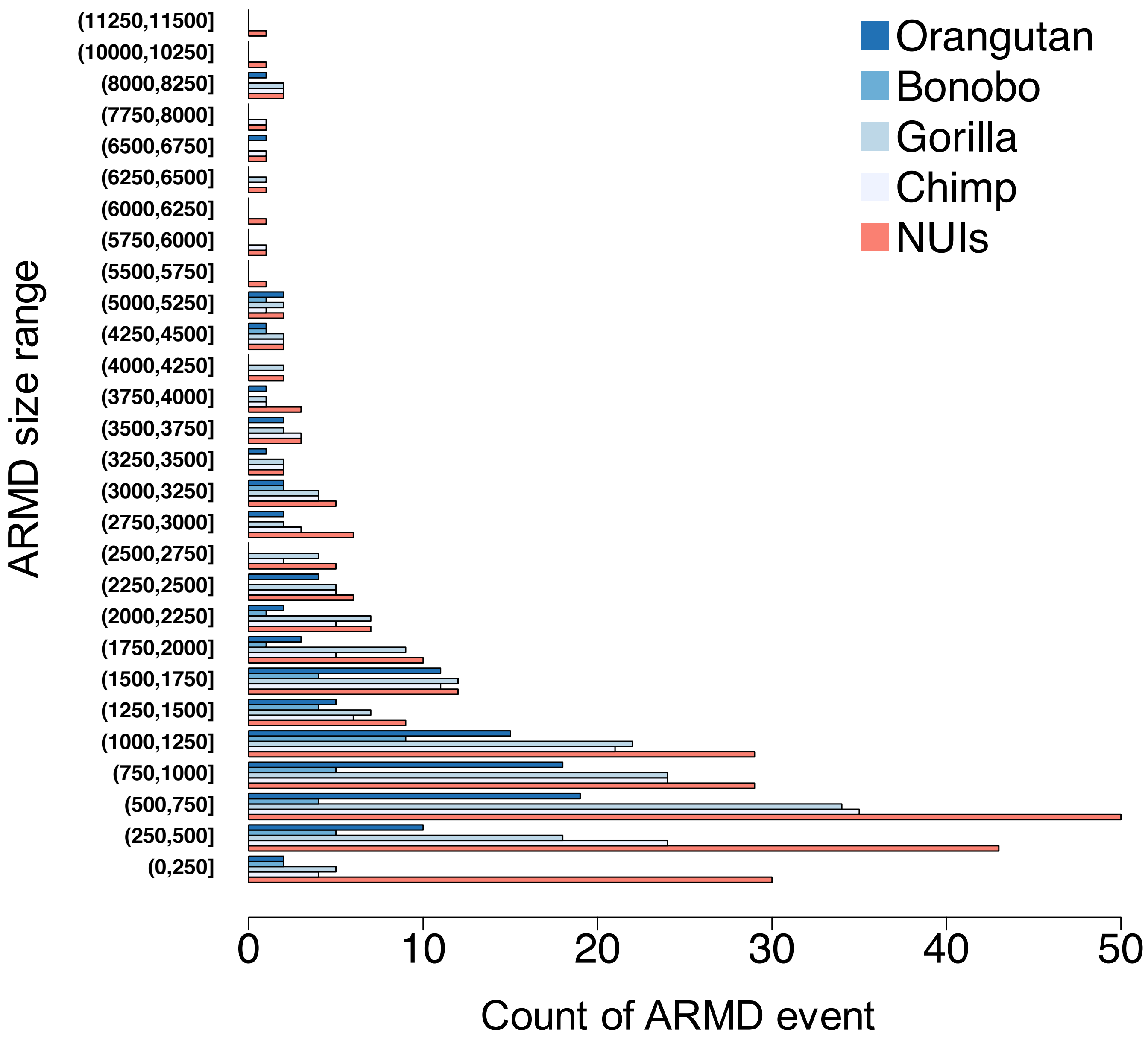

Supplementary Figure 3: *Alu* recombination-mediated deletion size distributions. Horizontal histogram illustrating the ARMD count identified in the 17 human samples and the four non-human primate genomes. A total of 265 events, depicted in pink, were observed in our data set. The other four bars in different shades of blue represent the ARMD events found in the chimpanzee genome (163 events), the gorilla genome (167 events), the bonobo genome (102 events), and the orangutan genome (38 events).

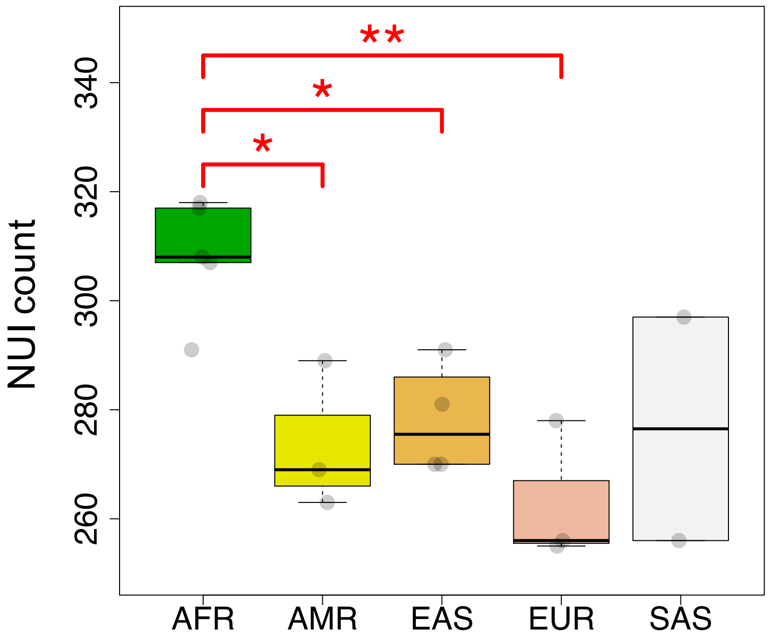

Supplementary Figure 4: Population structure of NUIs shared with the Icelandic group. The number of shared NUIs across the five populations groups. Each grey dot shows the actual NUI number per individual. ANOVA $F(4,12) = 6.216$; $p = 0.006$; followed by Tukey [AFR-AMR] $p = 0.0349966$; [AFR-EAS] $p = 0.0462222$; [AFR-EUR] $p = 0.0058295$. AFR – Africans; AMR – Americans; EAS – East Asians; EUR – Europeans; SAS – South Asians. The box plot illustrates the median, the upper and lower quartiles for each population. The whiskers correspond to the 1.5X interquartile range, or if no points exceed that distance, they refer to the minimum and maximum values in each group. $*$ $p \leq 0.05$; $**$ $p \leq 0.01$.

SUPPLEMENTARY TABLES

Supplementary Table 1: Summary statistics of the 17 genome assemblies

Sample	Super population	Population	Population Description	Molecule N50 (kb)	Distance between het sites (kb)	Phased block N50 (Mb)	Contig N50 (kb)	Scaffold N50 (Mb)	No. of scaffolds over 10kb	Assembly size (Gb)
**HG00250**	EUR	GBR	BRITISH FROM ENGLAND AND SCOTLAND, UK	88.4	1.6	2.5	126	18.1	1,547	2.8
**HG00353**	EUR	FIN	FINNISH IN FINLAND	95.8	1.6	2.7	122	16.8	1,240	2.7
**HG00512**	EAS	CHS	HAN CHINESE SOUTH, CHINA	103.0	1.7	2.7	113	15.3	1,333	2.7
**HG00733**	AMR	PUR	PUERTO RICAN IN PUERTO RICO	106.5	1.5	3.4	124	18.1	1,207	2.7
**HG00851**	EAS	CDX	CHINESE DAI IN XISHUANGBANNA, CHINA	103.3	1.6	2.7	129	18.6	1,119	2.7
**HG01971**	AMR	PEL	PERUVIAN IN LIMA, PERU	98.0	1.5	3.1	124	18.0	1,297	2.8
**HG02623**	AFR	GWD	GAMBIAN IN WESTERN DIVISION, THE GAMBIA	112.7	1.2	8.1	118	15.4	1,316	2.7
**HG03115**	AFR	ESN	ESAN FROM NIGERIA	105.0	1.2	8.5	122	17.6	1,311	2.7
**HG03838**	SAS	STU	SRI LANKAN TAMIL IN THE UK	102.3	1.5	3.6	120	23.5	1,228	2.7
**NA18552**	EAS	CHB	HAN CHINESE IN BEIJING, CHINA	106.3	1.5	2.8	121	16.1	1,285	2.7
**NA19068**	EAS	JPT	JAPANESE IN TOKYO, JAPAN	49.0	1.6	1.2	119	15.6	1,396	2.7
**NA19240**	AFR	YRI	YORUBA IN IBADAN, NIGERIA	124.8	1.1	9.3	119	16.3	1,168	2.7
**NA19440**	AFR	LWK	LUHYA IN WEBUYE, KENYA	98.5	1.1	8.1	125	19.2	1,217	2.7
**NA19789**	AMR	MXL	MEXICAN ANCESTRY IN LOS ANGELES, CALIFORNIA, USA	115.5	1.4	4.8	123	19.5	1,370	2.7
**NA19921**	AFR	ASW	AFRICAN ANCESTRY IN SOUTHWEST USA	159.4	1.2	14.8	113	21.2	1,198	2.8
**NA20587**	EUR	TSI	TOSCANI IN ITALIA (TUSCANS IN ITALY)	88.9	1.5	2.3	121	14.7	1,410	2.8
**NA21125**	SAS	GIH	GUJARATI INDIANS IN HOUSTON, TEXAS, USA	95.0	1.4	2.6	127	17.0	1,323	2.7

Supplementary Table 2: Validation of Non-reference unique insertions using BioNano insertion call set

Sample	Number of validated variants (≥2kb)	Total number of variants (≥2kb)	Precision rate (%)
**HG00250**	62	73	84.9%
**HG00353**	59	67	88.1%
**HG00512**	53	63	84.1%
**HG00733**	52	63	82.5%
**HG00851**	60	65	92.3%
**HG01971**	56	64	87.5%
**HG02623**	61	70	87.1%
**HG03115**	65	69	94.2%
**HG03838**	60	67	89.6%
**NA18552**	75	78	96.2%
**NA19068**	63	75	84%
**NA19240**	67	77	87%
**NA19440**	72	79	91.1%
**NA19789**	57	66	86.4%
**NA19921**	57	65	87.7%
**NA20587**	52	60	86.7%
**NA21125**	66	71	93%

Supplementary Table 3: Overall repeat content of Non-reference unique insertions

Repeat elements	Percentage of sequence
**SINEs:**	**21.45%**
ALUs	19.15%
MIRs	2.26%
**LINEs:**	**23.41%**
LINE1	19.88%
LINE2	3.07%
L3/CR1	0.34%
**LTR elements**	**10.66%**
ERVL	2.03%
ERVL-MaLRs	2.94%
ERV_classI	4.65%
ERV_classII	0.51%
**DNA elements:**	**3.01%**
hAT-Charlie	1.36%
TcMar-Tigger	1.05%
**Unclassified:**	**0.90%**
**Small RNA:**	**0.05%**
**Satellites:**	**0.53%**
**Simple repeats:**	**2.65%**
**Low complexity:**	**0.21%**

SUPPLEMENTARY NOTES

The following information explains the non-reference unique insertion variant naming format as seen in **Supplementary Data 1** and **Supplementary Data 2**.

NUI variant naming format: chromosome:breakpoint1-breakpoint2

If an NUI is flanked by homologous sequences on both ends, breakpoint1 will be larger than breakpoint2. In that case, the sequence between breakpoint1 and breakpoint2 is repeated twice, flanking the NUI on both ends (See below diagram). The yellow block represents the NUI while the flanking blue blocks represent the homologous sequences.

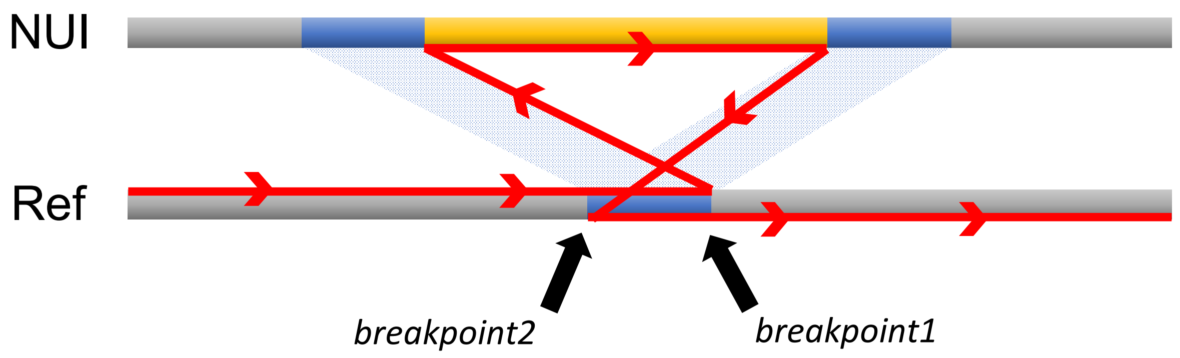

Supplement: Supplementary file 1 — Supplementary Information [file 41467_2018_5513_MOESM1_ESM.pdf]
