# Supplementary material for: De novo human genome assemblies reveal spectrum of alternative haplotypes in diverse populations
Source: Nat Commun. 2018 Aug 2;9:3040. doi: 10.1038/s41467-018-05513-w (PMC6072799; doi:10.1038/s41467-018-05513-w)
Supplement: Supplementary file 3 — Description of Additional Supplementary Files [file 41467_2018_5513_MOESM3_ESM.pdf]

Description of Additional Supplementary Files

File Name: Supplementary Data 1
Description: NUI sequences in FASTA format

File Name: Supplementary Data 2
Description: NUI occurrence matrix
